# Genome analysis of the kiwifruit canker pathogen *Pseudomonas syringae* pv. *actinidiae* biovar 5

**DOI:** 10.1038/srep21399

**Published:** 2016-02-19

**Authors:** Takashi Fujikawa, Hiroyuki Sawada

**Affiliations:** 1 NARO Institute of Fruit Tree Science, National Agriculture and Food Research Organization (NARO), Fujimoto 2-1, Tsukuba, Ibaraki 305-8605, Japan; 2 National Institute of Agrobiological Sciences, Kannondai 2-1-2, Tsukuba, Ibaraki 305-8602, Japan

## Abstract

*Pseudomonas syringae* pv. *actinidiae* (Psa) is a destructive pathogen of kiwifruit bacterial canker disease, causing severe economic losses to kiwifruit industry worldwide. Biovar 5 is the most recently reported biovar of Psa, and is found in only a local area of Japan at present. There is not much information of genetic characteristics of biovar 5. Thus, the genome of biovar 5 was sequenced and analyzed to clarify its detailed genetic characteristics. Here, the genomes of strain MAFF 212056 and MAFF 212061 of biovar 5 were estimated to be about 6.3 Mbp and 6.5 Mbp, respectively, and their phylogenetic positions were proved to be near that of biovar 2 in the phylogenetic tree. However, it was confirmed that biovar 5 had neither the coronatine biosynthetic genes conserved in biovar 2, its phylogenetic neighbor, nor the phaseolotoxin biosynthetic genes conserved in biovar 1, Japanese native pathogen. In addition, 45 genes of type III secreted effectors were identified in biovar 5 genomes, showing that their composition is different from that in the other biovars. Moreover, some biovar 5-specific regions were identified. Then, biovar 5-specific PCR primers for targeting these regions were designed, and proved to be applicable for detecting biovar 5 specifically.

Kiwifruit is the most economically important member of the genus *Actinidia*, and is cultivated worldwide. Green-fleshed *Actinidia deliciosa* and yellow- or red-fleshed *Actinidia chinensis* are well known species commercially cultivated. Production of kiwifruit is increasing because of consumer demand for its good taste and nutrition. However, recently, the spread of kiwifruit canker disease has been observed worldwide, which caused severe economic losses, in some cases limiting the cultivation of kiwifruit^1^.

Kiwifruit canker disease is caused by *Pseudomonas syringae* pv. *actinidiae* (Psa), and it was first identified in Japan, 1984^2^. Infected vines exhibit leaf spots, cane wilting, cane die-back, and canker, with abundant production of a red or milky-white exudate. Besides Japan, Psa has also been found in major production areas of the world. At first, these strains derived from diverse origins were divided into five biovars (biovars 1 to 5) on the basis of genetic diversity and toxin productivity^3^,^4^. Thereafter, biovar 4 was separated from Psa and assigned to another pathovar, which leads to the present situation that there remain four biovars (biovars 1, 2, 3 and 5) in Psa^5^. In brief, biovar 1, which includes the first identified Japanese strains of Psa, can produce phaseolotoxin; biovar 2 is found in only South Korea and can produce coronatine instead of phaseolotoxin; biovar 3 is a pandemic virulent group found worldwide and does not produce any known toxins; “biovar 4”, a low virulent group found in New Zealand, Australia, and France, had been transferred to the new pathovar *actinidifoliorum* (Psaf)^5^, which is now divided into four lineages (lineages 1 to 4) (lineages in Psaf are considered to be equivalent to biovars in Psa)^5^; and biovar 5 was recently found in Japan, 2012^4^,^6^. Both biovar 1 and biovar 3 have been found to be widely distributed in the kiwifruit cultivation area of Japan^2^,^6^. In contrast, biovar 5 is found only in a limited local area (Saga Prefecture located on Kyushu situated most southwesterly of Japanese four main islands)^4^, showing that this biovar may be endemic at present. However, there is no guarantee that it remains endemic in the future.

Sawada *et al.*^4^ have reported that biovar 5 does not produce both phytotoxins phaseolotoxin and coronatine and is rather related to biovar 2 than the major domestic group biovar 1 based on multilocus sequence analysis (MLSA). However, the genetic background of pathogenicity and physiology for biovar 5 is still unclear. Thus, we determined the genome sequences of biovar 5 and performed comparative genome analysis to clarify the detailed characteristics of biovar 5. Here, we have reported the genetic relationship among Psa biovars at a genomic level and new findings for the genes involved in host interaction and biovar 5-specific markers for diagnosis.

## Results

### Genome information of biovar 5

The genomic DNAs of strains MAFF 212056 (http://www.gene.affrc.go.jp/databases-micro_search_detail_en.php?maff=212056) and MAFF 212061 (http://www.gene.affrc.go.jp/databases-micro_search_detail_en.php?maff=212061), which were isolated from Saga Prefecture in 2012, were sequenced as representative biovar 5 strains. By using the next generation sequencer Ion PGM system (Life Technologies, Thermo Fisher Scientific Inc., Waltham, MA), 3,835,257 single reads of an average length of 189.9 bp, and 6,425,035 single reads of an average length of 189.4 bp were obtained respectively in MAFF 212056 and MAFF 212061. Genome *de novo* assembly was performed using the CLC Genomics Work bench (CLC Bio, Qiagen, Valencia, CA). After filtering with a Phred score cutoff of ≤20, 291 contigs of >500 bp with an N_50_ of 50,639 bp in MAFF 212056 and 687 contigs of >500 bp with an N_50_ of 50,026 bp in MAFF 212061 were obtained (Suppl. Doc 1 and 2). Consequently, the genome size (the assembled sequence size) of MAFF 212056 was estimated to be 6,342,665 bp, corresponding to a 126.4-fold genome coverage and that of MAFF 212061 was estimated to be 6,499,176 bp, corresponding to a 173.8-fold genome coverage. The both genomes have G + C content of 58.5%. The assembled data of MAFF 212056 were subjected to DDBJ MiGAP, and 6,330 protein-coding sequences (CDSs), 4,055 ribosomal binding sites, 55 tRNA genes, and 4 rRNA genes were identified. The sequences of some housekeeping genes of MAFF 212056, such as *acnB*, *cts*, *gapA*, *gyrB*, *pfk*, *pgi*, and *rpoD*, and 16S-23S rDNA ITS, which have been widely used as markers for phylogenetic analysis, were confirmed to be identical to those of MAFF 212061 and also to those of reported in previous studies^4^,^7^. Because the entire sequences of these housekeeping genes could be secured from our genome data, and the number of CDSs (6,330) predicted from our genome data proved to correspond to those of Psa (between 5,200 and 6,520) predicted from published genome database (e.g., 5,259 CDS, 5,761 CDSs, and 6,520 CDSs in the case of ICMP 9617, ICMP 18884 and ICMP18886, respectively)^8^, we have decided that our genome data have enough quality for further analysis.

### ANI analysis

DNA-DNA hybridization (DDH) experiments have been performed to determine genetic similarity between bacteria since the 1960s. Even if there is no information on DNA sequences, DDH experiment can reveal the similarity between compared bacteria. However, nowadays, genomic sequences can be acquired easily, so DDH *in silico* using genomic sequences has become more practical than conventional DDH experiment. As the representative of such *in silico* analyses, the average nucleotide identity (ANI) assay is often used^9^. The ANI value is calculated as the mean of identity by BLASTn matches between the virtually fragmented query genome and the reference genome. Hence, ANI can be used as a good tool for phylogenetic analysis at the genomic level. After obtaining the genomic sequences of biovar 5 in this study, ANI analysis of all Psa biovars including biovar 5 became possible for the first time.

**Figure 1:**
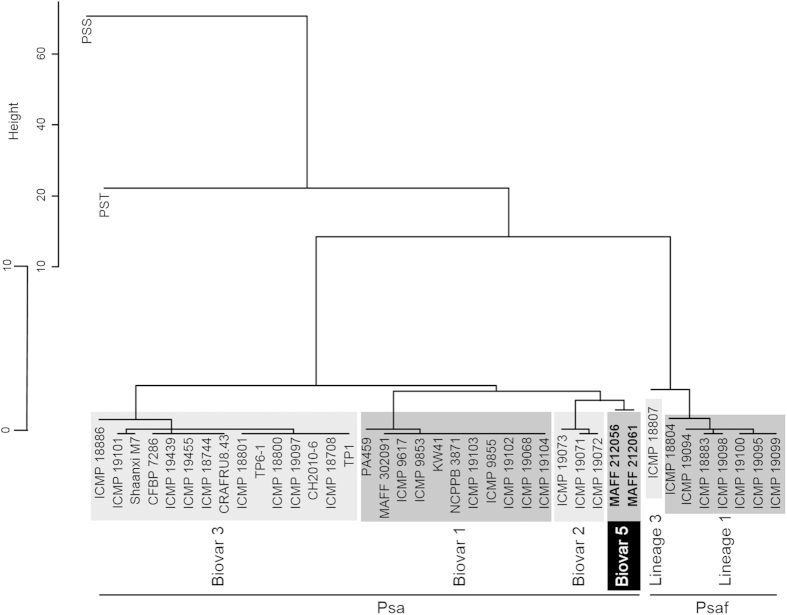
Dendrogram for ANI values. ANI value for each combination between strains was calculated, and a dendrogram was constructed using UPGMA. *Pseudomonas syringae* pv. *syringae* B728a (PSS) and *P. s.* pv. *tomato* DC3000 (PST) were used as outgroups. Psa “biovar 4” had been transferred to the new pathovar *actinidifoliorum* (Psaf) with four lineages (lineages 1 to 4)^5^, of which lineage 1 and lineage 3 were included in this analysis (lineages in Psaf are considered to be equivalent to biovars in Psa).

ANI values among Psa biovars were obtained using ANI calculator analysis (Suppl. Table 1), showing that the value between strains MAFF 212056 and MAFF 212061 of biovar 5 was approximately 100%. Then, a dendrogram of relatedness by using ANI values was constructed on the basis of UPGMA, which shows that strains MAFF 212056 and MAFF 212061 clustered tightly and independently of the other Psa biovars, among which biovar 2 was the nearest neighbor to biovar 5 (Fig. 1). Of the existing Psaf lineages (lineages 1 to 4), lineage 1 and lineage 3 were included in this analysis, which grouped together and were entirely separated from Psa biovars 1, 2, 3 and 5. Similar topologies were obtained when other algorithms (complete-linkage clustering method, nearest-neighbor chain algorithm method, and ward method) were used (data not shown).

### Toxins and effector genes

The results of ANI analysis (Fig. 1) and MLSA^4^ indicate that biovar 5 is the nearest neighbor to biovar 2. However, unlike biovar 2, *cfl* gene of the coronatine gene cluster was not amplified from biovar 5 DNA by using PCR^4^. In addition, some components of the *tox* island, a genomic island which contains *argK-tox* cluster (phaseolotoxin synthesis gene cluster)^10^, i.e., *amtA*, *desl* (*ptx*), *argD* (ORF3), and *argK*, conserved in most biovar 1 strains^4^,^10^,^11^, were also not amplified from biovar 5^4^. The type III secreted effector (T3SE) genes, whose translated products are translocated to host plant cells via the type III secretion system, are involved in virulence and/or avirulence^12^. However, the comprehensive composition of T3SE genes for biovar 5 has not yet been clarified. Thus, comparative genome analysis using the Mauve tool and virulent genes search by tBLASTx were performed to confirm the absence of these toxin genes and predict the effector genes in biovar 5 genomes.

**Figure 2:**
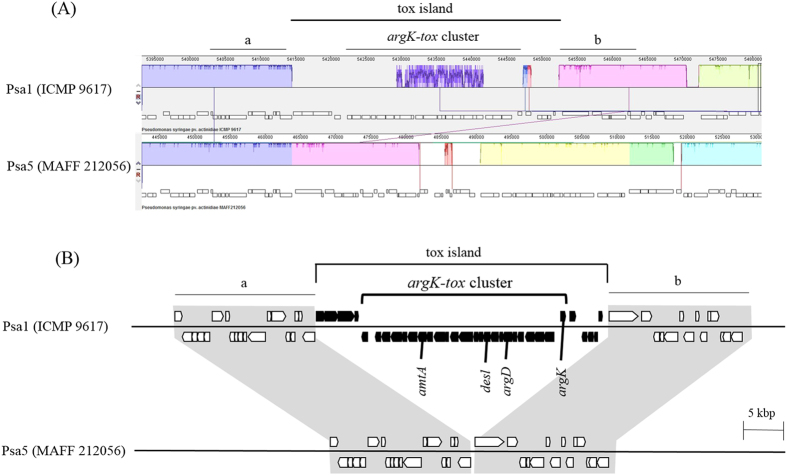
Comparative analysis of biovar 1 and biovar 5. Results of the comparative genome analysis by using the Mauve tool (**A**) and their schematic illustration (**B**) are presented. Locus of the *tox* island, a genomic island which contains *argK-tox* cluster (phaseolotoxin synthesis gene cluster)^10^, and its flanking regions (indicated by (**a**,**b**)) in biovar 1 (upper column), and the corresponding locus in biovar 5 (lower column) are shown. Box arrows indicate predicted genes and their directions. Black box arrows (components of *tox* island) are conserved in only biovar 1 and absent in biovar 5. Only the upstream region (**a**) and downstream region (**b**) of *tox* island in the biovar 1 genome were conserved continuously in the corresponding site of the biovar 5 genome.

When compared with the biovar 1 genome, no homologs of the *tox* island including *argK-tox* cluster were found in the homologous site of the MAFF 212056 genome, and only the flanking regions of the *tox* island in the biovar 1 genome were conserved continuously in the MAFF 212056 genome (Fig. 2). The phaseolotoxin markers, *amtA*, *desl* (*ptx*), *argD* (ORF3), and *argK* of the *argK-tox* cluster^4^,^10^, were entirely absent in the whole of the MAFF 212056 genome. In addition, since the ordering and orientation of contigs could be inferred using the Mauve tool, the contigs of MAFF 212056 was conveniently concatenated and a single draft genome of MAFF 212056 could be generated. Comparing a draft genome of MAFF 212056 with the biovar 1 genome (ICMP 9617) using the Artemis comparison tool (ACT), it was found that overall structure of the MAFF 212056 genome generally resembled that of biovar 1, but that only the latter possessed the *tox* island containing *argK-tox* cluster (Suppl. Fig. 1). Similarly, no homologs of the *tox* island containing *argK-tox* cluster were found in the MAFF 212061 genome of biovar 5 (data not shown).

**Figure 3:**
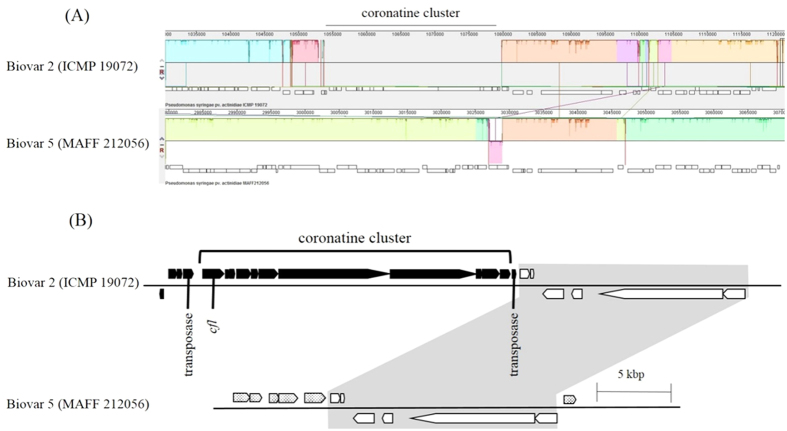
Comparative analysis of biovar 2 and biovar 5. Results of the comparative genome analysis by using the Mauve tool (**A**) and their schematic illustration (**B**) are presented. Locus of the coronatine gene cluster and its neighboring region in biovar 2 (upper column), and the corresponding locus in biovar 5 (lower column) are shown. Box arrows indicate predicted genes and their directions. Black box arrows (coronatine gene cluster) are conserved in only biovar 2 and absent in biovar 5. Because the coronatine gene cluster in biovar 2 was located on the edge of a contig in the biovar 2 draft genome, only its downstream region in the biovar 2 genome is shown in upper column of Panel (**B**). This downstream region was also conserved in the biovar 5 genome, being flanked by unrelated genes (dotted box arrows).

Next, when compared with the biovar 2 genome, no homologs of the coronatine gene cluster were found in the MAFF 212056 genome of biovar 5 (Fig. 3). Also, MAFF 212061 genome were found to possess no homologs of the coronatine gene cluster (data not shown).

Further, by comparisons among the Psa genomes using the Mauve tool and/or tBLASTx, 12 T3SE genes following *hopAB3* listed in Table 1 were newly found in Psa genomes in this study, in addition to 51 T3SE genes, which were reported previously as the total number of T3SE genes of Psa^8^. The total of 63 T3SE genes predicted in this study in conjunction with the previous report^8^ were highly homologous to the corresponding genes of ICMP 9617 (a biovar 1 strain) and ICMP 18884 (a biovar 3 strain), whose genomes were completely sequenced (Suppl. Table 2).

The 17 T3SE genes, which were predicted to be conserved in all the other Psa biovars (biovars 1, 2, 3) and Psaf by McCann *et al.*^8^, were also found to be present in the biovar 5 genomes. In total, the 45 T3SE genes were predicted to be located in the biovar 5 genomes (Table 1). Their composition was confirmed to be identical between MAFF 212056 and MAFF 212061 genomes. Comprehensively, it seems that the set of T3SE genes in biovar 5 resembled the set in biovar 1 or biovar 2.

**Figure 4:**
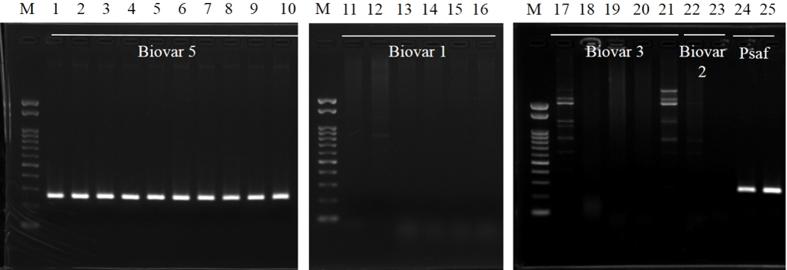
PCR analysis for *hopL1* possession in Psa. M; 0.1–2-kb Marker, 1–10; biovar 5 (1, MAFF 212054; 2, MAFF 212055; 3, MAFF 212056; 4, MAFF 212057; 5, MAFF 212058; 6, MAFF 212059; 7, MAFF 212060; 8, MAFF 212061; 9, MAFF 212062; 10, MAFF 212063); 11–16, biovar 1 (11, MAFF 211985; 12, MAFF 211986; 13, MAFF 302093; 14, MAFF 302145; 15, MAFF 302966; 16, MAFF 613024); 17–21, biovar 3 (17, MAFF 212101; 18, MAFF 212104; 19, MAFF 212107; 20, MAFF 212116; 21, MAFF 212117); 22–23, biovar 2 (22, ICMP 19072; 23, ICMP 19073); and 24–25, Psaf (Psa “biovar 4”) (24, ICMP 18804; 25, ICMP 18807). The amplicons of *hopL1* were obtained in both biovar 5 and Psaf strains.

On the basis of tBLASTx, *hopL1* was predicted to be present only in biovar 5 and Psaf. Thus, to further confirm whether Psa biovars and Psaf possess *hopL1* or not, PCR analysis was performed using Psa genomic DNA as templates with the *hopL1-*specific primers designed in this study (Table 2). As a result, a single band was obtained from the DNAs of biovar 5 and Psaf (Fig. 4), whereas no band was obtained in the case of DNA from biovars 1, 2, and 3, which endorsed the prediction of our comparative genomic analysis.

### Biovar 5-specific primers

**Figure 5:**
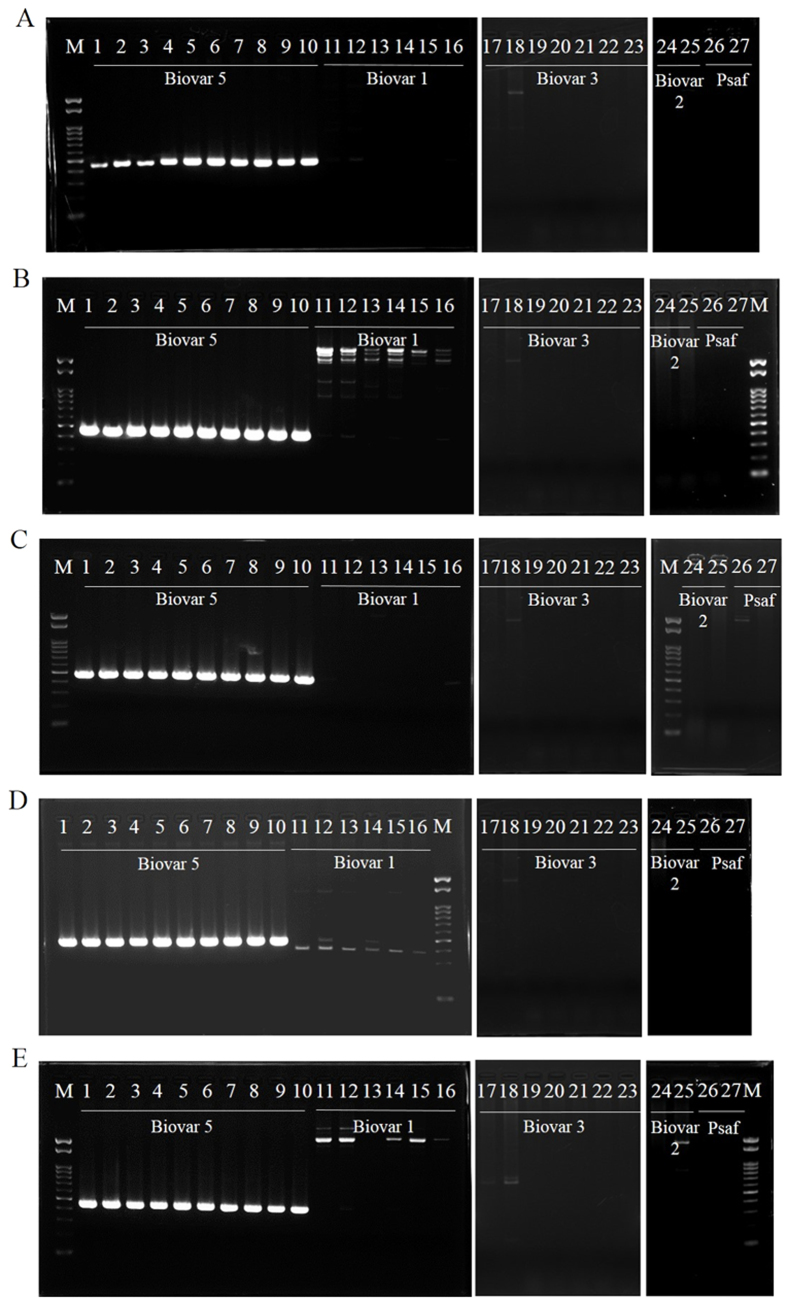
PCR analysis for biovar 5-specific sequences. Con002 (**A**), Con034 (**B**), Con044 (**C**), Con047 (**D**), and Con067 (**E**). M, 0.1–2-kb Marker; 1–10, biovar 5 (1, MAFF 212054; 2, MAFF 212055; 3, MAFF 212056; 4, MAFF 212057; 5, MAFF 212058; 6, MAFF 212059; 7, MAFF 212060; 8, MAFF 212061; 9, MAFF 212062; 10, MAFF 212063); 11–16; biovar 1 (11, MAFF 211985; 12, MAFF 211986; 13, MAFF 302093; 14, MAFF 302145; 15, MAFF 302966; 16, MAFF 613024); 17–23, biovar 3 (17, MAFF 212101; 18, MAFF 212104; 19, MAFF 212107; 20, MAFF 212116; 21, MAFF 212117; 22, MAFF 212121; 23, MAFF 212122); 24–25, biovar 2 (24, ICMP 19072; 25, ICMP 19073); and 26–27, Psaf (Psa “biovar 4”) (26, ICMP 18804; 27, ICMP 18807). The proper amplicons from each target region were obtained in only biovar 5 strains.

It may be possible to use *hopL1* as a biovar 5 marker; however, this marker cannot discriminate between biovar 5 and Psaf. Thus, to detect only biovar 5 certainly, biovar 5-specific sequences, whose highly homologous regions were not found in biovars 1, 2, 3, Psaf, Pss B728a, Pst DC3000, and *P. s.* pv. *phaseolicola* 1448A, were sought using comparative genomic analysis with the Mauve tool. Then, appropriate loci from only five contigs of MAFF 212056 (Contig_002, Contig_034, Contig_044, Contig_047, and Contig_067) were obtained (Suppl. Table 3), and biovar 5-specific primers were designed on the basis of their sequences (Table 2). All primer sets were confirmed to induce biovar 5-specific amplification by using PCR (Fig. 5). Electrophoresis showed the amplicons derived from biovar 5 templates to be single bands. However, Con047F/Con047R primers, targeting the internal sequence of Contig_047, induced nonspecific bands for biovar 1 templates. The size of the nonspecific bands derived from biovar 1 was close to that of the true amplicon from biovar 5 (Fig. 5D). Thus, four primer sets, except Con047F/Con047R primers (Table 2), proved to be applicable to biovar 5-specific detection.

## Discussion

Biovar 5 strains of Psa were found in 2012 for the first time, and their genetic diversity, toxin productivity, and various phenotypic characteristics were elucidated^4^. This biovar was found in only a local area (Saga Prefecture) of Japan, at the time of June 2015^6^. Thus, biovar 5 may be a domestic or endemic pathogen of kiwifruit and other relevant species. By contrast, biovar 3, a pandemic pathogen, could cause a global outbreak^1^,^3^,^8^. Genotypic characteristics of the known Psa biovars except biovar 5 have been investigated in detail^1^,^5^,^6^,^8^,^10^,^11^, whereas there is lesser information on biovar 5. Therefore, we cannot clearly explain the differences or similarity among the biovars, including biovar 5. In this study, we performed genome sequencing and comparative analysis to obtain detailed genetic information on biovar 5.

The representative strains MAFF 212056 and MAFF 212061 of biovar 5 are found to have genome size of ca. 6.3–6.5 Mbp and more than 6000 CDSs by using genome assembly and gene annotation. In addition, ANI analysis, which corresponds to DDH *in silico* analysis, indicates that the phylogenetic position of biovar 5 is close to that of biovar 2 at the whole-genome level (Fig. 1). A phylogenetic tree of all biovars was constructed using MLSA of seven housekeeping genes, showing that biovar 5 strains were placed adjacent to biovar 2 strains^4^. Thus, ANI analysis results obtained in this study are consistent with the MLSA results. However, biovar 5 does not possess the coronatine gene cluster, unlike biovar 2 (Fig. 3). As for T3SE composition, biovar 5 is different from the other biovars, including biovar 2 (Table 1). Moreover, biovar 5 was found to differ from biovar 2 with respect to biochemical characteristics, for example, biovar 5 can hydrolyze aesculin, produce strong fluorescent pigment on King’s B medium, and cannot utilize D-mannitol^4^. These results support the conclusion of Sawada *et al.*^4^ that biovar 5 should be regarded as an independent group separated from biovar 2. Hereafter, much effort including genome sequencing and phenotypic characterization of more strains of biovar 5 and biovar 2 will be necessary for unambiguous definition of biovar circumscription.

The *tox* island containing *argK-tox* cluster is conserved in most biovar 1 strains^4^,^10^,^11^, and the coronatine gene cluster is considered to be conserved in biovar 2 strains^1^; however, biovar 5, unlike biovars 1 and 2, is proved to have none of these toxin gene clusters. Thus, it is assumed that biovar 5 may produce unknown virulence factors including toxins. In fact, we have estimated that a locus in Contig_047 of MAFF 212056, which is a biovar 5-specific region, might be involved in the synthesis of some secondary metabolites. At this locus, a gene cluster ca. 20 kb, which contains some enzyme genes catabolizing fatty acids and polyketides, was found. This composition of the gene cluster resembled that of other toxin gene clusters such as coronatine^13^. Certainly, further studies are required to confirm whether this region is involved in novel toxin production.

For many plant pathogenic bacteria, not only toxins but also various effectors involved in host interaction are essential to establish infection and cause diseases. Various genes that encode secreted proteins and secretion apparatus, such as the type I, II, III, IV, and VI secretion system, were found in the biovar 5 genome. Their functions and roles need to be studied in the future.

Of various secreted proteins, T3SEs have been the most noted targets. T3SEs are significant proteinous factors for host interaction, and they work as virulence factors in susceptible hosts or as avirulence factors in resistant hosts with corresponding resistant genes^12^. The functions *in planta* and biophysical and biochemical actions of many T3SEs, especially from *Pseudomonas* spp., have been analyzed^12^. Consequently, the roles of T3SEs in *Pseudomonas* pathogens have become revealed considerably. By analogy with them, Psa is also assumed to use T3SEs for the establishment of infection in kiwifruit. Here, by comparative genome analysis in conjunction with the results of McCann *et al.*^8^, 63 T3SE genes were confirmed in Psa biovars in total, out of which, forty-five genes were found in the biovar 5 genomes (Table 1). These genes are highly homologous to the known corresponding T3SE genes of other biovars and species.

Here, the homolog of *hopL1* was found in only biovar 5 and Psaf (Table 1), which was predicted to be homologous to *hopL1* of *Pseudomonas avellanae* BPIC 631, a causal pathogen of bacterial canker in hazelnut (*Corylus avellana* L.), by using MiGAP annotation and tBLASTx. In addition, genome sequence information showed that some *Pseudomonas* plant pathogens (e.g., *P. cichorii* JBC1, *P. syringae* pv. *tomato* DC3000, and *P. s.* pv. *syringae* B728a) possess the *hopL1* homologs. However, the function of the HopL1 protein in host plants is unknown. Field survey and inoculation test have suggested that the pathogenicity of biovar 5 and Psaf may be inferior to that of biovars 1, 2, and 3^4^,^5^. Thus, it is assumed that *hopL1* may be related to the reduction in pathogenicity of biovar 5 and Psaf. Meanwhile, in the biovar 5 genomes, the *avrD1* homolog gene was found (Table 1). Because *avrD* in various *Pseudomonas* pathogens encodes the syringolide family glycolipid elicitor, which induces gene-for-gene resistance to host plants^14^, biovar 5 may have the ability for syringolide-inducible host interaction. Hereafter, function of these T3SEs of biovar 5 should be investigated further on a genetic and physiological basis.

We designed five biovar 5-specific primer sets (Table 2). Of these primer sets, at least four sets (i.e., Con002F/R, Con034F/R, Con044F/R, and Con067F/R) are proved to be applicable for biovar 5-specific detection. When diagnosis to examine whether biovar 5 strains exist in samples such as pollen or other plant organs is required, plant quarantine inspectors or researchers will be able to perform PCR by using any of these primer sets. Psa is proved to be highly heterogeneous and divided to biovars 1, 2, 3, and 5, whose virulence against kiwifruit is quite different one another^1^,^4^,^5^,^6^. Thus, a clear distinction is required in the field and quarantine for implementing control measures quickly and efficiently. Biovar 1-specific primers^11^, biovar 2-specific primers^15^, and biovar 3-specific primers^6^,^16^ have been developed, in addition to all Psa-universal primers^17^,^18^. Therefore, the biovar 5-specific primers developed in this study (Table 2) are essential to complement the diagnostic procedure of kiwifruit bacterial canker disease. Hereafter, biovar 5 can be specifically detected by using these primer sets, and prompt disease control can be implemented.

## Methods

### Bacterial strains, culture, and DNA extraction

The bacterial strains and their relevant characteristics are listed in Table 3. Bacteria were routinely cultured on Luria agar plates or potato dextrose agar plates at 27 °C. Genomic DNA from bacteria were extracted using the InstaGene Matrix (Bio-Rad, Hercules, CA) or DNeasy Plant mini kit (Qiagen), according to the manufacturer’s instructions.

### Genome sequencing and annotation

The strains MAFF 212056 (http://www.gene.affrc.go.jp/databases-micro_search_detail_en.php?maff=212056) and MAFF 212061 (http://www.gene.affrc.go.jp/databases-micro_search_detail_en.php?maff=212061) were chosen as the representative biovar 5 strains. These genomic DNAs were processed to template samples by using the Ion Plus Fragment Library Kit and Ion PGM Template OT2 400 Kit with Ion OneTouch 2 System (Life Technologies, Thermo Fisher Scientific Inc.). Then, the template samples of biovar 5 were sequenced using the Ion Sequencing 400 Kit and a 318 Chip with the next generation sequencer Ion PGM (Life Technologies, Thermo Fisher Scientific Inc.). Sequence data were assembled and analyzed using the CLC Genomic Work Bench (CLC Bio, Qiagen). The genome sequence of biovar 1 strain ICMP 9617 (GenBank accession number: CM002753) was used as the reference for mapping and assembly of sequence reads.

The assembled contigs of MAFF 212056 were annotated using the DDBJ Microbial Genome Annotation Pipeline (MiGAP) (http://www.migap.org/index.php/en) and edited manually for entry into the nucleotide sequence databases (DDBJ/ENBL/GenBank).

### Genomic average nucleotide identity

The concatenated contig sequences of biovar 5 were compared with reference sequences to estimate the genetic distance among biovar 5, the other Psa biovars, and Psaf. Average nucleotide identity (ANI) analysis, which is used for *in silico* analysis of DNA-DNA hybridization (DDH)^9^, was used. ANI values of combinations between the whole genome sequences of Psa and Psaf strains were calculated using a web tool, ANI calculator (http://enve-omics.ce.gatech.edu/ani/). The matrix made from ANI values between Psa and Psaf strains was converted to a genetic dendrogram with algorithms such as the unweighted pair group method with arithmetic mean (UPGMA) and complete-linkage clustering method (farthest neighbor clustering method) via R program. ICMP 9853 (GenBank accession number: ANJB00000000), ICMP 9855 (AOKB00000000), ICMP 19068 (AOJX00000000), ICMP 19102 (AOKA00000000), ICMP 19103 (AOJQ00000000), ICMP 19104 (AOJZ00000000), KW41 (AGNP00000000), PA459 (AGNQ00000000), MAFF 302091 (AEAL00000000), ICMP 9617 (AFTH00000000) and NCPPB 3871 (AFTF00000000) from biovar 1 strains, ICMP 19071 (AOJS00000000), ICMP 19072 (AOJW00000000) and ICMP 19073 (AOJR00000000) from biovar 2 strains, CFBP 7286 (AGNO00000000), CH2010–6 (AGUH00000000), ICMP 18708 (ANJC00000000), ICMP 18744 (ANGD00000000), ICMP18800 (ANJD00000000), ICMP 18801 (AOKQ00000000), ICMP 19097 (AOKN00000000), ICMP 19101 (AOKM00000000), ICMP 19439 (ANJM00000000), ICMP 19455 (ANJK00000000), CRAFRU8.43 (AFTG00000000), Shaanxi_M7 (ANJJ00000000), TP1 (ANJG00000000) and TP6–1 (ANJH00000000) from biovar 3 strains, ICMP 18804 (ANJE00000000), ICMP 18883 (AOKH00000000), ICMP 19094 (AOKJ00000000), ICMP 19095 (AOKI00000000), ICMP 19098 (AOKE00000000), ICMP 19099 (AOKD00000000), and ICMP 19100 (AOKC00000000) from Psaf (lineage 1) strains; ICMP 18807 (ANJL00000000) from Psaf (lineage 3) strains; and *P. s.* pv. *syringae* (Pss) B728a (CP000075) and *P. s.* pv. *tomato* (Pst) DC3000 (AE016853) from outer groups were used as reference genome sequences.

### Comparative genome analysis

To find the virulent genes and identify biovar 5-specific sequences, biovar 5 contigs were aligned with the following reference sequences: ICMP 9617 (CM002753) and ICMP 9855 from biovar 1 strains, ICMP 19072 and ICMP 19073 from biovar 2 strains, ICMP 18801 and ICMP 18884 (CM002751) from biovar 3 strains, ICMP 18804 from Psaf (lineage 1) strain, ICMP 18807 from Psaf (lineage 3) strain, and Pss B728a, Pst DC3000, and *P. s.* pv. *phaseolicola* 1448A (NC_005773) using Mauve, which is a multiple genome alignment tool (http://darlinglab.org/mauve/mauve.html)^19^. On the basis of the aligned sequences, highly homologous regions or solitary regions between biovar 5 and the references were observed, and biovar 5-specific sequences were collected. In addition, the ordering and orientation of contigs were estimated using the Mauve tool, and the concatenation of contigs of MAFF 212056 was performed to generate a virtual single genome sequence. Then, a draft genome of MAFF 212056 was compared with the biovar 1 genome (ICMP 9617) using the Artemis comparison tool (ACT) (http://www.sanger.ac.uk/science/tools/artemis-comparison-tool-act)^20^.

Toxin synthesis genes and type III secreted effector (T3SE) genes were considered as representative virulent genes, and the homologs of such virulent genes were identified by tBLASTx (E value < 1 e^−10^) using candidate CDSs obtained from biovar 5 genomes as query sequences against NCBI database. Moreover, according to the method of McCann *et al.*^8^, the homologs were also identified and/or confirmed by tBLASTx (E value < 1 e^−5^) using representative virulent genes obtained from a public database (http://pseudomonas-syringae.org) as query sequences against biovar 5 genomes. Partial hits or truncated/disrupted sequences were indicated as ‘incomplete’. The translational identity of T3SE genes present in biovar 5 genomes was confirmed by comparing with the corresponding genes of a biovar 1 strain (ICMP 9617) or a biovar 3 strain (ICMP 18884).

### Polymerase chain reaction

Primers used in this study are listed in Table 2. PCR was performed in a 20-μl reaction mixture with 1× PCR buffer containing 0.2 mM dNTPs, 0.25 μM of each primer, 0.5 U of *ExTaq* HS polymerase (Takara Bio Inc., Shiga, Japan), and 2 μl of PCR template DNAs. The PCR conditions were 9 min of pre-denaturation at 96 °C, followed by 35 cycles of 30 s of denaturation at 96 °C, 1 min of annealing at 55 °C, 30 s of extension at 72 °C, and then a single final extension of 7 min at 72 °C. The presence and amounts of PCR products were confirmed using agarose gel electrophoresis.

## Additional Information

**Accession codes:** The nucleotide sequence data of MAFF 212056 are available in the DDBJ/EMBL/GenBank database under accession numbers BBWG01000001–BBWG01000291.

**How to cite this article**: Fujikawa, T. and Sawada, H. Genome analysis of the kiwifruit canker pathogen *Pseudomonas syringae* pv. *actinidiae* biovar 5. *Sci. Rep.* **6**, 21399; doi: 10.1038/srep21399 (2016).

## Supplementary Material

Supplemental Document 1

Supplemental Document 2

Supplemental Table 1

Supplemental Figure 1, Table 2 and 3

## Figures and Tables

**Table 1:** Predicted type III secreted effector genes of biovar 5, the other Psa biovars and Psaf.

Genes^1^	biovar 5 (MAFF 212056)^2^	biovar 1^2^	biovar 2^2^	biovar 3^2^	Psaf (“biovar 4”)2,^3^
*avrRpm1*	incomplete	+	−	+	−
*avrE*	+	+	+	+	+
*hopM1*	+	+	incomplete	+	+
*hopAA1-1*	+	+	+	+	+
*hopN1*	+	+	+	+	+
*hopI1*	+	+	+	+	+
*hopS2*	+	+	+	+	+
*hopBB1-1*	−	variable	incomplete	+	−
*hopAO2*	−	variable	−	+	−
*hopAF1-1*	+	variable	variable	+	+
*hopBB1-2*	incomplete	variable	−	+	−
*hopAW1*	+	incomplete	−	+	−
*hopX3*	−	variable	+	+	−
*hopAY1*	+	variable	+	incomplete	+
*avrB4*	+	variable	+	+	−
*avrD1*	+	+	+	+	−
*hopD1*	+	+	+	+	−
*hopQ1*	+	+	+	+	−
*hopF2*	+	variable	+	+	−
*hopAR1*	−	variable	−	−	+
*hopF1*	−	−	−	−	+
*hopAF1-2*	incomplete	−	−	−	+
*hopA1*	+	−	incomplete	incomplete	+
*hopY1*	+	+	+	+	incomplete
*avrRpm2*	+	incomplete	+	incomplete	−
*hopZ3*	+	+	+	+	−
*hopAS1*	+	+	+	+	+
*hopZ5*	−	−	−	+	−
*hopH1*	−	−	−	+	−
*hopAM1-1*	−	incomplete	−	+	−
*hopAE1*	+	+	variable	+	+
*hopW1*	+	incomplete	incomplete	incomplete	+
*hopR1*	+	+	+	+	+
*hopAG1*	+	incomplete	+	+	incomplete
*hopAH1*	+	+	+	+	+
*hopAI1*	+	incomplete	+	+	+
*hopAM1-2*	+	−	−	+	−
*avrPto5*	+	+	+	+	−
*hopAZ1*	+	+	+	+	+
*hopAV1*	incomplete	variable	−	incomplete	−
*hopAA1-2*	−	−	−	incomplete	−
*hopAU1*	+	+	+	+	−
*hopX1*	−	variable	−	−	+
*hopX2*	−	variable	−	−	+
*hopBD2*	−	+	incomplete	−	−
*hopH3*	−	+	−	−	−
*hopO1*	−	−	−	−	+
*hopT1*	−	−	−	−	+
*hopS1*	−	−	−	−	+
*hopE1*	−	−	−	−	+
*hopAB3*	−	−	−	−	+
*eop3*^4^	+	+	+	+	−
*hopAH2-1*^4^	+	+	+	+	+
*hopAH2-2*^4^	+	+	+	+	+
*hopAJ2*^4^	+	+	+	+	+
*hopAK1*^4^	+	+	+	+	+
*hopAN1*^4^	+	+	+	+	+
*hopJ1*^4^	+	+	+	+	+
*hopL1*^4^	+	−	−	−	+
*hopP1*^4^	+	+	+	+	+
*hopPma1*^4^	+	+	+	+	+
*hopZ3*^4^	+	+	+	+	−
*hopAC1*^4^	+	+	+	+	+

^1^Type III secreted effector genes were predicted using the DDBJ MiGAP tool and tBLASTx.

^2^Partial hits or truncated/disrupted sequences were indicated as ‘incomplete’. Also, referring to McCann *et al.*^8^, when the presence/absence of a gene is dependent on strains of the same biovar, it was indicated as ‘variable’.

^3^Psa “biovar 4” had been transferred to the new pathovar *actinidifoliorum* (Psaf)^5^.

^4^Twelve genes following *hopAB3* are T3SE genes that were newly found in this study.

**Table 2:** Primers used in this study.

Name	Sequence (5′ to 3′)	Target	Amplicon size (bp)
Psa5-HopL1-F	TCAAACAGAGCGAAGTGGTG	Biovar 5-*hopL1*	214
Psa5-HopL1-R	CCCCATTGTTTCATCCAGTC		
Con002F	AACTCATACCCTGCGGTCAC	Biovar 5-specific region of Contig_002	449
Con002R	GACACCGAGCAAAACCAAAT		
Con034F	CCAAACAACGTCTGGGCTAT	Biovar 5-specific region of Contig_034	450
Con034R	TCGGCCTAGCTACGAGTGAT		
Con044F	AAGCGCCTTAATCTCGTTCA	Biovar 5-specific region of Contig_044	470
Con044R	ATTCCGGATTGGGTATCACA		
Con047F	GCTGCTCTCTGGGTACAAGG	Biovar 5-specific region of Contig_047	447
Con047R	ATCGAAGGTACGGTGGAGTG		
Con067F	ATTTTAACGCCCATCTGCAC	Biovar 5-specific region of Contig_067	439
Con067R	CTGCGGATTGCAACAGTCTA		

**Table 3:** Bacterial strains used in this study.

Strain	Biovar	Host plant	Location	Isolated year	Reference/Source
*Pseudomonas syringae* pv. *actinidiae* (Psa)					
MAFF 212054	5	*A. chinensis* ‘Hort16A’	Saga, Japan	2012	4
MAFF 212055	5	*A. chinensis* ‘Hort16A’	Saga, Japan	2012	4
MAFF 212056	5	*A. chinensis* ‘Hort16A’	Saga, Japan	2012	4
MAFF 212057	5	*A. chinensis* ‘Hort16A’	Saga, Japan	2012	4
MAFF 212058	5	*A. chinensis* ‘Hort16A’	Saga, Japan	2012	4
MAFF 212059	5	*A. chinensis* ‘Hort16A’	Saga, Japan	2012	4
MAFF 212060	5	*A. chinensis* ‘Hort16A’	Saga, Japan	2012	4
MAFF 212061	5	*A. chinensis* ‘Hort16A’	Saga, Japan	2012	4
MAFF 212062	5	*A. chinensis* ‘Hort16A’	Saga, Japan	2012	4
MAFF 212063	5	*A. chinensis* ‘Hort16A’	Saga, Japan	2012	4
MAFF 211985	1	*A. deliciosa*	Ehime, Japan	2000	NIAS Genebank
MAFF 211986	1	*A. deliciosa*	Ehime, Japan	2001	NIAS Genebank
MAFF 302093	1	*A. deliciosa*	Kanagawa, Japan	1985	NIAS Genebank
MAFF 302145	1	*A. deliciosa*	Wakayama, Japan	1988	NIAS Genebank
MAFF 302966	1	*A. deliciosa*	Niigata, Japan	1993	NIAS Genebank
MAFF 613024	1	*A. deliciosa*	Shizuoka, Japan	1995	NIAS Genebank
Saga-2	3	*A. chinensis* ‘Hort16A’	Saga, Japan	2014	This study
MAFF 212101	3	*A. chinensis* ‘Hort16A’	Saga, Japan	2014	6
MAFF 212104	3	*A. chinensis* ‘Rainbow Red’	Ehime, Japan	2014	6
MAFF 212107	3	*A. chinensis* ‘Rainbow Red’	Wakayama, Japan	2014	6
MAFF 212116	3	*A. deliciosa* ‘Hayward’	Fukuoka, Japan	2014	6
MAFF 212117	3	*A. deliciosa* ‘Hayward’	Fukuoka, Japan	2014	6
MAFF 212121	3	*A. chinensis* ‘Hort16A’	Saga, Japan	2014	6
MAFF 212122	3	*A. chinensis* ‘Hort16A’	Saga, Japan	2014	6
ICMP 19072	2	*A. chinensis*	Jeonnam, Korea	1997	8
ICMP 19073	2	*A. chinensis*	Jeonnam, Korea	1998	8
*Pseudomonas syringae* pv. *actinidifoliorum* (Psaf)*					
ICMP 18804	Lineage 1	*A. chinensis*	Te Puke, Bay of Plenty, New Zealand	2010–2011	8
ICMP 18807	Lineage 3	*A. deliciosa*	Tauranga, Bay of Plenty, New Zealand	2010–2011	8

*Psa “biovar 4” had been transferred to the new pathovar *actinidifoliorum* (Psaf)^5^, which is now divided into four lineages (lineages 1 to 4) (lineages in Psaf are considered to be equivalent to biovars in Psa).
