# Supplementary material for: Genome analysis of the kiwifruit canker pathogen Pseudomonas syringae pv. actinidiae biovar 5
Source: Sci Rep. 2016 Feb 19;6:21399. doi: 10.1038/srep21399 (PMC4759546; doi:10.1038/srep21399)
Supplement: Supplemental Figure 1, Table 2 and 3 [file srep21399-s4.pdf]

# Supplementary Figure 1. Whole genome comparative analysis of biovar 1 and biovar 5

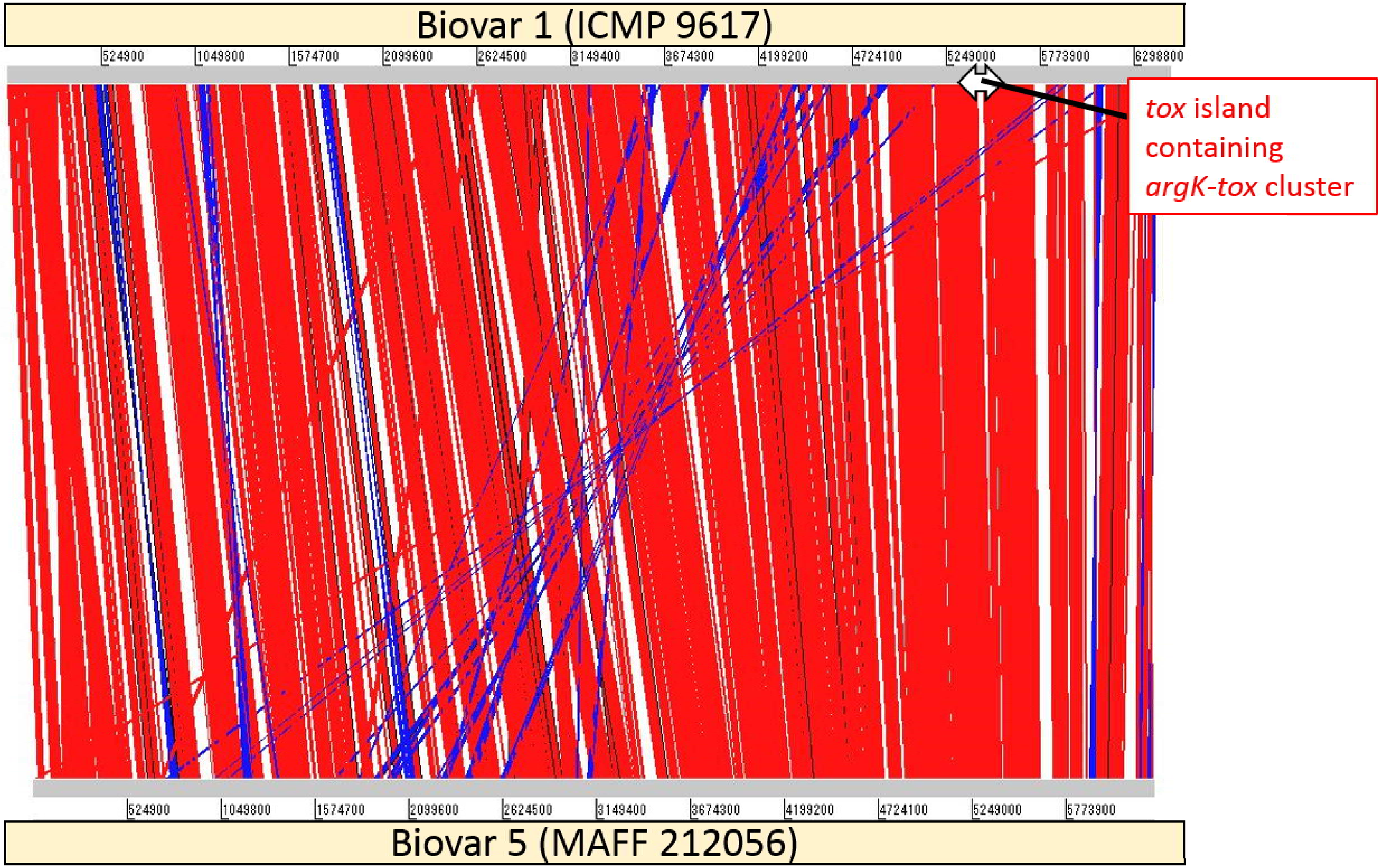


A result of the comparative genome analysis by using the ACT is presented. Upper bar is a genome sequence of biovar 1 (ICMP 9617), and lower bar is that of biovar 5 (MAFF 212056). The red and blue bands represent the forward and reverse matches, respectively. The two direction arrow, which is placed on the non-match region of biovar 1, corresponds to the region of the *tox* island containing *argK-tox* cluster in biovar 1 (Fig. 2), showing that the *tox* island is absent in biovar 5.

# Supplementary Table 2. Homology of T3SE genes

| | ICMP9617 (biovar 1) | ICMP18884 (biovar 3) |
|---|---|---|
| *avrE* | 99.71 | 99.71 |
| *hopM1* | 99.04 | 99.16 |
| *hopAA1-1* | 98.56 | 98.43 |
| *hopN1* | 100.00 | 100.00 |
| *hopI1* | 99.53 | 99.07 |
| *hopS2* | 100.00 | 100.00 |
| *hopAF1-1* | 100.00 | 99.53 |
| *hopAW1* | - | 100.00 |
| *hopAY1* | 99.63 | 100.00 |
| *avrB4* | 99.64 | 99.64 |
| *avrD1* | 99.64 | 99.68 |
| *hopD1* | 100.00 | 100.00 |
| *hopQ1* | 99.67 | 99.67 |
| *hopF2* | 99.03 | 99.03 |
| *hopA1* | - | 100.00 |
| *hopY1* | 98.92 | 98.92 |
| *avrRpm2* | 95.95 | 98.88 |
| *hopZ3* | 99.75 | 99.75 |
| *hopAS1* | 99.82 | 99.82 |
| *hopAE1* | 99.19 | 99.19 |
| *hopW1* | - | 98.81 |
| *hopR1* | 99.38 | 99.38 |
| *hopAG1* | - | 99.01 |
| *hopAH1* | 99.02 | 99.22 |
| *hopAI1* | - | 95.90 |
| *hopAM1-2* | - | 100.00 |
| *avrPto5* | 100.00 | 100.00 |
| *hopAZ1* | 100.00 | 100.00 |
| *hopAU1* | 99.77 | 98.98 |
| *eop3* | 100.00 | 100.00 |
| *hopAH2-1* | 100.00 | 100.00 |
| *hopAH2-2* | 100.00 | 100.00 |
| *hopAJ2* | 100.00 | 100.00 |
| *hopAK1* | 100.00 | 100.00 |
| *hopAN1* | 97.13 | 97.13 |
| *hopJ1* | 97.35 | 98.23 |
| *hopL1* | - | - |
| *hopP1* | 100.00 | 99.10 |
| *hopPma1* | 99.17 | 100.00 |
| *hopZ3* | 99.75 | 99.75 |
| *avrRpm2* | 95.95 | 98.88 |
| *hopAC1* | 100.00 | 99.73 |

The translational identity of T3SE genes present in biovar 5 genome was confirmed by comparing with the corresponding genes of a biovar 1 strain (ICMP 9617) or a biovar 3 strain (ICMP 18884).

# Supplementary Table 3. biovar 5-specific region of contigs

| No. | Contig number | Location of biovar 5-specific regions | Predicted proteins in this region | Left primer target | Left primer sequence (5'–3') | Right primer target | Right primer sequence (5'–3') | Amplified size |
|---|---|---|---|---|---|---|---|---|
| 1 | Contig_002 | 1–9050 | Hypothetical proteins (PSA5_00060–PSA5_00095) | 7802–7821 | AACTCATACCCTGCGGTCAC | 8231–8250 | GACACCGAGCAAAACCAAAT | 449 |
| 2 | Contig_034 | 1–24750 | Hypothetical proteins, Type IV secretion proteins, Stability protein, Single-stranded DNA binding protein, DNA topoisomerase proteins (PSA5_07155–PSA5_07310) | 20124–20143 | CCAAACAACGTCTGGGCTAT | 20554–20573 | TCGGCCTAGCTACGAGTGAT | 450 |
| 3 | Contig_044 | 19024–24488 | Hypothetical proteins (PSA5_08305–PSA5_8320) | 432–451 | AAGCGCCTTAATCTCGTTCA | 882–901 | ATTCCGGATTGGGTATCACA | 470 |
| 4 | Contig_047 | 44105–64666 | Hypothetical proteins, MutT, Major facilitator proteins, Arginine aminomutase, phosphopantetheine attachment site domain-containing protein, 3-oxoacyl-ACP synthase, non-ribosomal peptide synthetase, Beta-ketoacyl synthase cupin (PSA5_09455–PSA5_09535) | 3923–3942 | GCTGCTCTCTGGGTACAAGG | 4350–4369 | ATCGAAGGTACGGTGGAGTG | 447 |
| 5 | Contig_067 | 1–11428 | Hypothetical proteins, citrate transporter (PSA5_12530–PSA5_12585) | 1563–1582 | ATTTTAACGCCCATCTGCAC | 1982–2001 | CTGCGGATTGCAACAGTCTA | 439 |

Biovar 5-specific sequences, whose highly homologous regions were not found in biovars 1, 2, 3, Psaf, Pss B728a, Pst DC3000, and *P. s.* pv. *phaseolicola* 1448A, were sought using comparative genomic analysis with the Mauve tool. Then, appropriate loci from only five contigs (Contig_002, Contig_034, Contig_044, Contig_047, and Contig_067) were obtained. Also biovar 5-specific primers were designed on the basis of their sequences.
